# Generating random graphs with prescribed graphlet frequency bounds derived from probabilistic networks

**DOI:** 10.1371/journal.pone.0328639

**Published:** 2025-08-26

**Authors:** Bram Mornie, Didier Colle, Pieter Audenaert, Mario Pickavet

**Affiliations:** IDLab, Department of Information Technology, Ghent University – imec, Ghent, Belgium; Instituto Nacional de Medicina Genomica, MEXICO

## Abstract

Testing or benchmarking network algorithms in bioinformatics requires a diverse set of networks with realistic properties. Real networks are often supplemented by randomly generated synthetic ones, but most graph generative models do not take into account the distribution of subgraph patterns, i.e. *motifs* or *graphlets*. Moreover, in many cases, biological interactions are uncertain events and must be modeled by probabilistic graph edges. The uncertainty is often ignored in practice, which can lead to incorrect conclusions about the properties of biological networks. In this work, we instead derive bounds on the graphlet counts and degree distribution of a probabilistic target network and use this information as input to a novel random graph generation algorithm. The algorithm grows graphs incrementally by making small modifications in every step, which allows for an efficient graphlet counting method. Using this method, we can update graphlet counts after each iteration in a time independent of the total node number on sparse graphs. We evaluate our model on synthetic and real networks of different sizes and with different degrees of uncertainty. Although computation times strongly depend on the size of graphlets taken into account, our experiments demonstrate that graphs with over 10 000 edges and well-controlled frequencies of all three- and four-node graphlets can be generated in under an hour.

## 1 Introduction

Network theory is widely used to study complex systems in society. The system’s information is encoded in the topology of a graph and knowledge about the system is gained through graph-analytical methods. In biology, gene regulation, protein-protein interactions (PPI), metabolic pathways, etc. are often represented in the form of networks. For many years, researchers have used different kinds of random graphs to model real, experimentally observed networks in order to find non-random effects and discover the phenomena underlying a specific network topology [1–5]. Additionally, random graphs can be used to test or benchmark network-related algorithms when sufficient high-quality data is not readily available. As an example, a benchmark of genetic network inference methods is often performed on data generated from random graphs, as the number of *gold standard* networks is rather limited [6–9]. Especially in this application, a good match between the random graph and the real network is crucial. The continued importance of high-quality synthetic data was recently highlighted in [10].

Traditional random graph models aim to reproduce some general properties of real-life networks, such as long-tailed degree distributions [11] or small-worldness [12], but many topological intricacies are ignored. In particular, the occurrence and frequency of specific subgraph patterns, called *motifs* or *graphlets*, has been a highly active field of research since the seminal work of Shen-Orr *et al*. [13], yet none of the traditional models pay any attention to this. While some recently proposed graph generative models show a stronger focus on subgraph patterns [14, 15], a generator that allows full control over graphlet frequencies, is, to the best of our knowledge, not yet available.

Furthermore, the existence of edges in, e.g., gene association or PPI networks is not certain, because evidence of interactions is based on noisy experimental data and error-prone statistical models [16]. This uncertainty is often overlooked in network analysis, even though, as we show below, the characteristics of possible graphs sampled from a probabilistic model can be very different from those of the deterministic *backbone* graph.

In this work, we first extend an existing method to construct graphs with specific frequencies of small graphlets based on a deterministic network, to uncertain networks. Then, we introduce a novel algorithm, called GRAphlet-based Incremental generator for Probabilistic networks (GRAIP), for the generation of random graphs with graphlet frequencies and degree distributions similar to a probabilistic target network. GRAIP grows a graph in an incremental fashion while monitoring its properties. The step-by-step generation process allows for efficient incremental graphlet counting, making the counting problem tractable. In addition, our algorithm makes full use of the information about uncertainty to infer error margins on the degree distribution and graphlet frequencies of the target network. We then require that the properties of the generated graph lie within these margins. This approach makes the problem more manageable as opposed to demanding exact equality, while also producing a graph that is closer to reality, given the available information.

The remainder of this paper is organized as follows. In Sect 2, we discuss some preliminaries and background concepts regarding graphlets and uncertain graphs, needed for the following sections, and give a review of related work. We provide a detailed description of our algorithms in Sect 3. We experimentally evaluate our methods on synthetic networks, as well as real PPI networks, in Sect 4. Finally, we conclude in Sect 5.

## 2 Background

In this section, we first provide the preliminaries and notations needed to describe our methods (Sect 2.1). Then, we summarize the key literature related to graphlets and random graph models (Sect 2.2).

### 2.1 Preliminaries

#### 2.1.1 Deterministic graphs.

A deterministic graph is represented by the tuple G=(V,E), consisting of a node set $V=\{v_{1},...,v_{n}\}$ and an edge set E={(u,v)|u,v∈V}. The number of nodes, the order of the graph, is n=|V| and the number of edges, the size of the graph, is m=|E|. Only undirected graphs without self-loops and multi-edges are considered in this work. The degree histogram *N*_*G*_(*k*) is defined as the *number* of nodes of degree *k*, while the more commonly used degree distribution $P_{G}(k)=\frac{N_{G}(k)}{n}$ gives the *fraction* of nodes of degree *k*. In the next section, we will also make use of the clustering coefficient, which is a measure of the degree to which nodes tend to cluster together. The *global* clustering coefficient $CC_{g}$, is given by

$$CC_{g}=\frac{3T}{W+3T},$$ (1)

where *T* is the number of triangles (graphlet *M*_2_, see below) and *W* is the number of connected triplets that do not form a triangle (graphlet *M*_1_). The global clustering coefficient ranges between zero, for graphs with no triangles, and one, for complete graphs. On the other hand, the *local* clustering coefficient $CC_{l,v}$ for a node *v* is computed as:

$$CC_{l,v}=\frac{2t_{v}}{k_{v}(k_{v}-1)},$$ (2)

where $k_{v}$ is the degree of *v* and $t_{v}$ is the number of triangles that include *v*, which is also equal to the number of edges between neighbors of *v*. In a homogeneous graph, it holds that $\frac{1}{n}\sum_{v\in V}CC_{l,v}=CC_{g}$.

**Fig 1:**
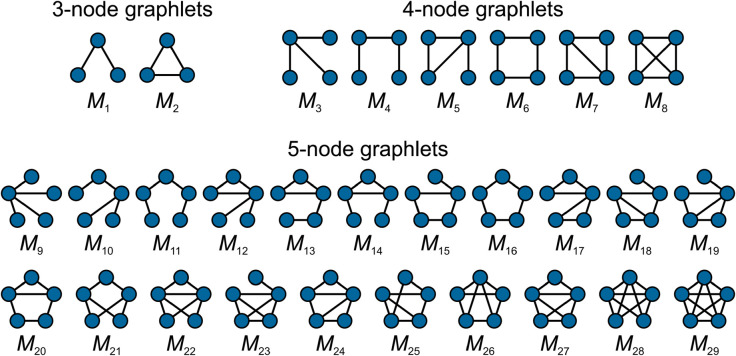
Undirected graphlets of orders three, four and five.

An induced subgraph $S=(V_{S},E_{S})$ of *G* is a subgraph satisfying $\forall u,v\in V_{S}:(u,v)\in E_{S}⟺(u,v)\in E$. A *graphlet* $M=(V_{M},E_{M})$ is defined as a connected, induced subgraph of a graph *G*. In the literature, usually graphlets of three to five nodes are considered [3, 17, 18], but this is not a strict limit. Fig 1 shows the 29 undirected graphlets with up to five nodes. The frequency or count of graphlet *M* in *G*, *C*_*M*,*G*_, is the number of distinct subgraphs of *G* that are isomorphic to *M*. Two subgraphs are distinct if they differ in at least one edge.

#### 2.1.2 Uncertain graphs.

An uncertain or probabilistic graph is a triplet 𝒢=(V,E,P), which, apart from the node and edge set, also includes a function P:E→(0,1] that assigns to each edge an existential probability. The probabilities *P*(*e*) are considered independent. The deterministic graph defined by *V* and *E* is called the *backbone graph* of 𝒢. *Possible World Semantics* is often used to represent 𝒢 as a probability distribution over a set {$G_{i}=(V,E_{i})|E_{i}\subseteq E$} of $2^{|E|}$ deterministic graphs [19]. In generating a possible graph *G*_*i*_, a particular edge e∈E is included with probability *P*(*e*) and excluded with probability (1–*P*(*e*)). The existential probability $P(G_{i})$ of the graph *G*_*i*_ is hence given by the following equation:

$$P(G_{i})=\prod_{e\in E_{i}}P(e)\prod_{e\in E⧵E_{i}}(1-P(e)).$$ (3)

Given an uncertain graph 𝒢, we wish to derive the mean E and variance Var of the degree distribution and graphlet frequencies in 𝒢. For an individual node v∈V, the mean and variance of its degree $k_{v}$ can easily be computed as

$$\text{E}(k_{v})=\sum_{e|v\in e}P(e),$$ (4)

$$\text{Var}(k_{v})=\sum_{e|v\in e}P(e)(1-P(e)).$$ (5)

However, in order to derive the statistical properties of the full degree distribution, the mean and variance of the number of nodes of degree *k* is needed. This requires the probability $P(k_{v}=k)$ that the degree of node *v* is equal to *k*, for every v∈V, which can be very expensive to compute: for every node *v* with degree $k_{v}^{\prime }$ in the backbone graph larger than or equal to *k*, we must enumerate all (kv′k) combinations of edges that lead to degree *k*.

**Fig 2:**
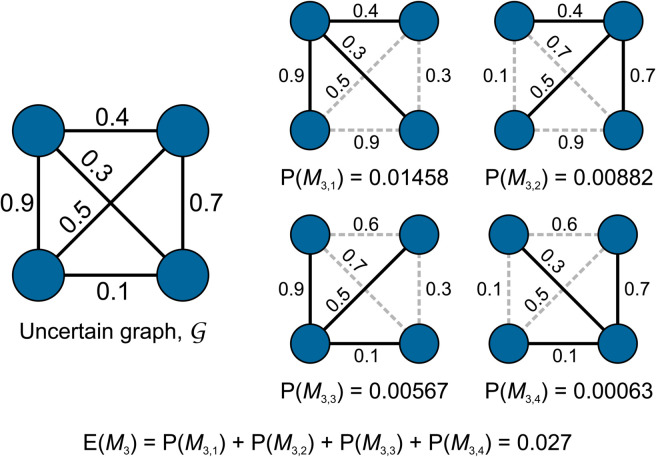
Expected number of star graphlets *M*_3_ in an uncertain complete graph of order 4. The uncertain graph contains four possible instances of *M*_3_, each with its own existential probability P(*M*_3,*i*_). The probability value next to the edges in the possible graphs is the value that must be used to compute the existential probability of that graph (*P*(*e*) if *e* is part of the graph, (1–*P*(*e*)) if it is not).

The situation is even more problematic for graphlet frequencies. Consider, for example, the uncertain graph on Fig 2. If this were a deterministic graph, it would contain a single instance of graphlet *M*_8_. Due to the uncertainty, the possible world of 𝒢 also includes every four-node graphlet of lower edge density, such as the star graphlet *M*_3_ counted on Fig 2. Hence, to derive the statistics of a certain graphlet type, we must also consider all graphlet types of higher density in the backbone graph. This quickly becomes computationally intractable on even moderately sized graphs.

### 2.2 Related work

#### 2.2.1 Graphlets.

Graphlets and motifs are often considered to be fundamental building blocks of a complex network. Shen-Orr *et al*. defined network motifs as frequent subgraph patterns that appear in frequencies much higher than those found in randomized networks [13]. Motif-based analysis attracted a lot of attention in network biology, among other fields, especially for graph classification [17, 20]. However, Pržulj *et al*. noted that infrequent patterns or patterns with average frequencies cannot be neglected in a full-scale network comparison and introduced graphlets as *any* small, connected, induced subgraph of a larger network [3, 21].

Determining graphlet or motif frequencies is a computationally intensive task. A lot of research has been devoted to the design of efficient counting algorithms, generally focusing on subgraphs of three to five nodes. Traditional approaches are based on enumeration of subgraph patterns [22–25]. More recently, authors have tried to speed up subgraph counting by avoiding a full enumeration through the use of combinatorial equations [26–29]. For example, Pinar *et al*. introduced the ESCAPE algorithm based on a cutting framework to systematically derive equations for efficient counting of four- and five-node non-induced subgraphs, which can easily be transformed into induced counts afterwards [29]. There also exist methods that estimate subgraph frequencies in large networks based on exact counts in smaller subnetworks. We do not go into detail, but refer the interested reader to a survey by Ribeiro *et al*. [30].

Some researchers have studied subgraph counting in uncertain graphs. In [31], the authors estimated the mean of motif counts in uncertain networks, but only considered the rather unrealistic case of uniform edge existence probabilities. Todor *et al*. proposed a method to calculate the mean and variance of motif frequencies in probabilistic biological networks [32]. They provide both an exact computation and a way to approximate the variance in larger networks. Ma *et al*. introduced both a simple sampling framework, PGS, and a more advanced version, LINC, that relies on strong similarities between samples to avoid restarting the count from scratch with every sample [33].

#### 2.2.2 Random graph generation.

The first random graph model considered graphs with a fixed number of nodes and either a fixed probability of an edge between any two nodes [34], or a fixed number of edges randomly distributed among the nodes [1]. This is the well-known Erdős-Rényi (ER) model. The ER model is very popular due to its simplicity, but many properties of realistic networks are not reproduced by ER graphs. Most notably, ER graphs have a binomial degree distribution, while heavy-tailed distributions are often observed in real-world networks. This observation lead to preferential attachment models, of which the best known example is the Barabási-Albert (BA) model [11]. Other well-known random graph models that try to mimic the properties of real-world networks, are the small-world model of Watts and Strogatz (WS) [12] and random geometric graphs [35].

While the above models have some parameters that can be tuned by the user, there is ultimately little direct control over the properties of the generated graphs. In contrast, the configuration model considers an ensemble of graphs with a fixed degree sequence [36]. It was developed for theoretical analysis of random graphs, but is also used to sample from prescribed degree sequences. In [37], Karrer and Newman extended the configuration model to random graphs containing specified frequencies of subgraphs. The authors provide an extensive theoretical analysis of the model, but its practical use is very limited. Exponential Random Graph Models (ERGMs) define a probability distribution over networks with given statistics [38]. The statistics can be chosen to capture various network properties, but parameter estimation is computationally demanding, especially for larger networks.

Bach *et al*. developed a graph generator based on an evolutionary algorithm to generate graphs similar to a target network [39]. Similarity is defined based on a variety of graph properties, but graphlets are not considered. In [14], an algorithm to generate networks with specific motifs is proposed. However, the approach only encourages the formation of certain motifs, but does not put actual constraints on motif frequencies. Mursa and Andreica recently introduced another evolutionary algorithm to generate networks with high assortativity degree and high local clustering coefficient [15], because they found that there is a significant positive correlation between these two properties and the occurrence of motifs [40]. They also put no direct constraints on motif counts.

Another class of graph generators is the deep generative models. As an example, GraphRNN is a deep autoregressive model for generating diverse graphs that match the structural characteristics of a target set [41]. In [42], Goyal *et al*. proposed GraphGen, a generator that can also deal with labeled graphs. Both GraphRNN and GraphGen were tested on their ability to reproduce automorphism orbit counts and perform well on this criterion, with GraphGen consistently showing the best performance [42]. A downside is that these models do not scale well beyond graphs with hundreds of nodes and edges. We could give many more examples of deep generative models, but instead refer the interested reader to the relevant literature [43].

An alternative to generating a graph from scratch is to start from a randomized version of a real network. In [17], such a randomized network is obtained by repeatedly swapping the endpoints of randomly chosen pairs of edges. In a second step, edges are again swapped at random, but this time, the frequencies of specific graphlet types are monitored and a simulated annealing technique [44] is used to converge to frequencies close to those in the real network. A very similar method is considered in [45]. In the next section, we extend this approach to uncertain target networks.

## 3 Materials and methods

Since the swapping method described in the previous requires a single deterministic graph to start from, it is not immediately applicable to probabilistic networks, which correspond to a distribution of deterministic graphs. In this section, we first propose a modified version of this algorithm and discuss its limitations (Sect 3.1). Then, we give a detailed description of our novel algorithm, GRAIP, of which the purpose is to incrementally generate a random graph with degree distribution and graphlet counts within margins dictated by a probabilistic target network (Sect 3.2).

Both algorithms require information on the statistical characteristics of certain graph properties. As we argued in Sect 2.1, it is very expensive to compute such characteristics exactly. Instead, we approximate the mean and variance of the degree distribution and graphlet counts through sampling. More concretely, a set of *N* possible graphs *G*_*i*_ is obtained from an uncertain network 𝒢 by Monte-Carlo sampling based on the existential probabilities *P*(*e*). Then, a graphlet counting algorithm for deterministic graphs (ESCAPE [29]) is used to calculate the graphlet frequency $C_{M,G_{i}}$ for each graphlet *M* and the exact degree distribution $P_{G_{i}}$ of *G*_*i*_ is computed as well. We considered the use of the more sophisticated LINC sampling algorithm by Ma *et al*. [33], but it was found to be far too memory-intensive when a large number of graphlets (e.g., all graphlets of order five or lower) have to be counted.

### 3.1 Swapping model for uncertain networks

The issue with the swapping model is that it is not clear which real graph should be randomized when working with uncertain networks. Two candidates are the backbone graph and a single possible graph *G*_*i*_, but in both cases, all information about uncertainty is effectively discarded. The goal of the randomization procedure is to obtain a graph with the same degree distribution as the real network, but that is completely random in every other aspect. The configuration model achieves the same goal and only requires a degree sequence to start from.

We therefore propose to combine the configuration model with the simulated annealing part of the swapping model. The resulting algorithm consists of the following steps (for more details, see [17, 36]):

*Step 1* Extract a degree sequence from the expected degree distribution, obtained through sampling.
*Step 2* Construct a random graph based on this degree sequence, following the configuration model.
*Step 3* Perform random edge swaps and use a simulated annealing method to obtain graphlet frequencies close to the expected ones.

The resulting graph has a degree distribution and graphlet frequencies close to the expected values, if the simulated annealing algorithm is fully converged. In the following, we will call this model SwapCon.

There are two major downsides to this approach. First, it does not scale well to larger graphs and/or graphlets, because graphlet counts have to be determined after every edge swap. As every swap is a rather local change, computation speed might be improved by using a counting algorithm that only considers subgraphs touching a certain edge. We use such an algorithm in GRAIP (see below), but we found that it is generally not faster, and often significantly slower, in SwapCon. The reason is that every swap is equivalent to two removed edges and two added edges, and hence the local search must be performed four times. For most graphs, it is more efficient to use a combinatorial algorithm like ESCAPE. The second downside is that no information about the spread of the uncertain graph’s properties is taken into account. The standard deviation on graphlet frequencies could be used to determine if the simulated annealing algorithm is converged, but the algorithm would still aim to reproduce the expected frequencies. Our new algorithm, discussed next, performs better in both aspects.

### 3.2 GRAIP

**Fig 3:**
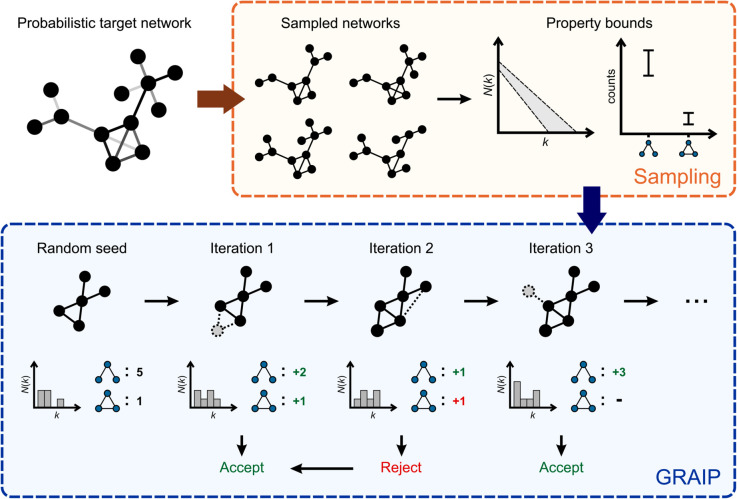
Workflow of the GRAIP method.

The GRAIP algorithm is described in Algorithm 1. Fig 3 provides a visualization of the general workflow. The generator function takes as input an uncertain graph 𝒢 and six parameters: *S*, $n_{g}$, *max*_*s*_, node_step, *w* and *max*_*rej*_. We call the generated deterministic graph *H*. The first parameter, *S*, defines the number of possible graphs sampled for the estimation of the statistical properties of the degree distribution and graphlet counts of 𝒢. We consider all graphlets of order at most $n_{g}$, e.g., if $n_{g}=5$, all graphlets of three, four and five nodes are taken into account. In some works, an edge is seen as the sole graphlet of order two. However, we do not explicitly put a constraint on the number of edges, but rather assume that this number is already more or less fixed by the other constraints. The iterative generation process is stopped when either the properties of the current graph lie within the predetermined bounds, or the number of iterations reaches *max*_*s*_. The last three parameters will be explained in more detail below.

**Algorithm 1. Incremental graph generator.**

**Input:** Uncertain graph 𝒢, number of samples *S*, maximum graphlet order $n_{g}$, maximum number of steps *max*_*s*_, number of steps between addition/removal of a node node_step, weight factor w∈[0,1] for cost function, maximum number of rejected changes before a guaranteed accepted change *max*_*rej*_

**Output:** Generated graph $H=(V_{H},E_{H})$

1: **GraphGenerator**(𝒢, *S*, $n_{g}$, *max*_*s*_, node_step, *w*, *max*_*rej*_)

2: $E_{n}$, $s_{n}$, $E_{m}$, $s_{m}$, ${𝐄}_{N}$, ${𝐬}_{N}$, ${𝐄}_{c}$, ${𝐬}_{c}$ ← Sample(𝒢, *S*, $n_{g}$)

3: bins, weights ← BinDegrees(${𝐄}_{N}$, *S*)       ⊳ See Algorithm 3

4: ${𝐄}_{p}\leftarrow \frac{weights}{E_{n}}$

5: ${𝐬}_{p}\leftarrow \frac{Histogram({𝐬}_{N},bins)}{E_{n}}$

6: $\overset{―}{CC_{g}}\leftarrow$ average global clustering coefficient in 𝒢

7: H← BA graph with $n=|V_{H}|=\frac{E_{n}}{5}$, $m=|E_{H}|=\frac{E_{m}}{5}$

8: $P_{H}\leftarrow$ degree distribution of *H*

9: $C_{H}\leftarrow$ list of counts of order $\leq n_{g}$ graphlets in *H*

10: **for** *step* = 1,...,*max*_*s*_ **do**

11:   **if** $P_{H}\in [{𝐄}_{p}-2{𝐬}_{p},{𝐄}_{p}+2{𝐬}_{p}]$ **and** $C_{H}\in [{𝐄}_{c}-2{𝐬}_{c},{𝐄}_{c}+2{𝐬}_{c}]$ **then**

12:            ⊳ meaning, e.g., $P_{H,i}\in [E_{p,i}-2s_{p,i},E_{p,i}+2s_{p,i}],\forall i$

13:    **return** *H*

14:   **end if**

15:   r← random number in [0,1]

16:   **if** step mod node_step=0 **then**

17:    $d_{n}\leftarrow \frac{n-E_{n}}{s_{n}}$

18:    **if** $r>\frac{1}{1+\exp(-d_{n})}$ **then**       ⊳ Add node

19:     $u,E_{u}\leftarrow$ AddNode(*H*, $\overset{―}{CC_{g}}$)

20:    **else**       ⊳ Remove node

21:     u← random element of $V_{H}$

22:    **end if**

23:   **else**

24:    $d_{m}\leftarrow \frac{\frac{m}{n}E_{n}-E_{m}}{s_{m}}$

25:    **if** $r>\frac{1}{1+\exp(-d_{m})}$ **then**       ⊳ Add edge

26:     u,v← random nodes in *H* for which edge $(u,v)\notin E_{H}$

27:    **else**       ⊳ Remove edge

28:     (u,v)← random element of *E*_*H*_

29:    **end if**

30:   **end if**

31:   $P_{T},C_{T}\leftarrow$ update $P_{H}$ and $C_{H}$ assuming the previously selected update is executed

32:   $cost_{H}\leftarrow$ Cost(Histogram($P_{H},bins$), $C_{H}$, ${𝐄}_{p}$, ${𝐬}_{p}$, ${𝐄}_{c}$, ${𝐬}_{c}$, *w*)       ⊳ See Algorithm 2

33:   $cost_{T}\leftarrow$ Cost(Histogram($P_{T},bins$), $C_{T}$, ${𝐄}_{p}$, ${𝐬}_{p}$, ${𝐄}_{c}$, ${𝐬}_{c}$, *w*)

34:   **if** $cost_{T}<cost_{H}$ **or** *T* was rejected *max*_*rej*_ times in a row **then**

35:    Update *H*, *n* and *m* by adding or removing the correct node *u* and/or edge(s) (*u*,*v*)

36:   **end if**

37: **end for**

38: **return** *H*

39:

40: **AddNode**(*H*, $\overset{―}{CC_{g}}$)

41: u← new node

42: v← pick a $v_{i}\in V_{H}$, according to weights $k_{v_{i}}$

43: **if** *v* is part of a clique of order ≥4 **then**

44:   $E_{u}\leftarrow$ list of edges $(u,c_{v}),\forall c_{v}\in$ LargestClique(*v*)

45: **else**

46:   $e_{nv}\leftarrow$ number of edges between neighbors of *v*

47:   $p_{nb}\leftarrow \frac{(k_{v}+1)\overset{―}{CC_{g}}}{2}-\frac{e_{nv}}{k_{v}}$

48:   Add edge $(u,n_{v}),n_{v}\in$ Neighbors(*v*) to $E_{u}$ with probability *p*_*nb*_

49: **end if**

50: **return** *u*, $E_{u}$

The algorithm starts by computing the mean E and standard deviation $s=\sqrt{Var}$ of the number of nodes ($E_{n}$, $s_{n}$) and edges ($E_{m}$, $s_{m}$), the degree histogram (${𝐄}_{N}$, ${𝐬}_{N}$) and the graphlet frequencies (${𝐄}_{c}$, ${𝐬}_{c}$) in 𝒢. We use boldface to distinguish symbols denoting lists of values from symbols denoting a single value. The lists ${𝐄}_{N}$ and ${𝐬}_{N}$ are binned (see below) and converted to the mean ${𝐄}_{p}$ and standard deviation ${𝐬}_{p}$ on the degree distribution. The allowed range for the properties $P_{H}$ and $C_{H}$ of the newly generated graph *H* is set to [𝐄−2𝐬,𝐄+2𝐬]. This interval includes most (95% if a normal distribution is assumed) of the sampled graphs. In Sect 2.1, it was stated that all possible graphs contain the full node set *V*, and hence $s_{n}$ should be zero. However, we want to allow some variability in *n* and therefore take the largest connected component in each sample. It is possible that a sample graph consists of two or several equally sized disconnected components, such that a significant part of the network is lost through the previous operation. In practice, this was rarely observed. The parts that are disconnected from the main component, are rather single nodes or groups of a few nodes.

As GRAIP grows a graph in an iterative fashion, some seed graph is required. If the seed is sufficiently small compared to the possible graphs of 𝒢, its exact topology has little to no influence on the final product. Here we opt for a BA graph with 20% of the nodes and edges. A small BA graph is a plausible choice due to the scale-free nature of the degree distribution commonly observed in biological networks. In the iterative generation process, a step can either consist of adding or removing a node, along with edges linked to this node, or adding or removing an edge. A possible change to *n* is made every node_step steps, with node_step≥1. It makes sense to choose node_step strictly greater than 1, as, e.g., removing a node also removes several edges and is hence a more significant change to the graph than removing a single edge. We found that node_step=5 works well in practice. Whether to add or remove a node is decided only partially at random. A function *f*_*n*_ is defined based on the current number of nodes *n* in *H*, $E_{n}$ and $s_{n}$:

$$f_{n}=\frac{1}{1+\exp\left(-\frac{n-E_{n}}{s_{n}}\right)}.$$ (6)

A random number r∈[0,1] is rolled and a node is added if *r*>*f*_*n*_, otherwise a node is removed. The logistic function *f*_*n*_ always returns a value between zero and one and has a desirable S-shape. It ensures that *n* is likely to be brought closer to $E_{n}$ if the deviation from $E_{n}$ is large. As an example, in the “growing” phase, where $\frac{n-E_{n}}{s_{n}}\ll 0$, $f_{n}\approx 0$ and it is very likely that *r*>*f*_*n*_. If $n=E_{n}$, it is equally likely for a node to be added or removed. In case a node is removed, an element from $V_{H}$ is chosen entirely at random, but the algorithm to add a node is more advanced. Denoting the new node by *u*, we first select an element *v* of $V_{H}$ that will certainly be a neighbor of *u*. The probability of selecting a specific node is proportional to its current degree, mimicking preferential attachment models. We found that this results in faster graph construction for networks with heavy-tailed degree distributions, such as PPI networks, as opposed to uniform probabilities. If *v* is part of a clique, i.e. a subgraph that is complete, of order at least four, *u* is also made part of the clique by adding edges between *u* and all the current members of the clique. This is to promote the formation of larger cliques, which are sometimes present in biological networks and are otherwise very unlikely to form during graph generation. If *v* is not part of an order ≥ 4 clique, edges between *u* and neighbors of *v* are added with a probability *p*_*nb*_ chosen such that the local clustering coefficient $CC_{l,v}$ will be close to the average global clustering coefficient $\overset{―}{CC_{g}}$ of 𝒢. The latter is derived from the mean counts of graphlets *M*_1_ and *M*_2_. Denoting by $k_{v}$ the degree of *v before* adding *u* and by $e_{nv}$ the number of edges between neighbors of *v*, the expected local clustering coefficient after adding edge (*u*,*v*) and edges $\left(u,n_{v}),\forall n_{v}\in Neighbors(v\right)$ with probability *p*_*nb*_ is

$$CC_{l,v,expected}=\frac{2(e_{nv}+p_{nb}k_{v})}{k_{v}(k_{v}+1)}.$$ (7)

If we also set $CC_{l,v,expected}=\overset{―}{CC_{g}}$, we find the expression for *p*_*nb*_ in Algorithm 1, line 47. To decide whether an edge should be added or removed, a similar criterion as for nodes is used. The main difference is that *m* is scaled by $\frac{E_{n}}{n}$. Otherwise, graphs with *n* significantly above or below $E_{n}$ would automatically be pushed towards lower, respectively higher, density. Which edge to add or remove is selected randomly. Finally, a change is accepted if the temporary graph *T* has a lower *cost*, defined by Algorithm 2, than the current graph *H*, or if *max*_*rej*_ changes in a row have been rejected. This last criterion was added to prevent the generation process from getting stuck when *H* almost has the desired properties. A simulated annealing approach was tested as well, but we found that either too many *bad* graphs were accepted, or none at all. The appropriate magnitude of *max*_*rej*_ depends on the size of the input graph. For graphs with at least a couple hundred edges, $max_{rej}=0.02E_{e}$ is a decent choice.

The cost function is described in Algorithm 2. It includes two contributions: one from the error on the degree distribution and another from the error on graphlet frequencies. The relative weight of the two contributions is controlled by the parameter *w*. It was observed that $w=\frac{2}{3}$ generally results in both contributions being more or less equally important.

For the first part of the total cost, the cumulative degree distribution $P_{G}^{c}$ is computed. The *k*’th element of $P_{G}^{c}$ is the number of nodes of degree at least *k*. The first contribution is the average relative deviation of $P_{G}^{c}$ from its mean in the target network. The cumulative degree distribution is used to avoid penalizing an excess of low-degree nodes, as long as there is a corresponding shortage of high-degree nodes. This approach is essential because, in our generator, high-degree nodes grow from low-degree nodes. For the graphlet counts, a logarithmic contribution seems more appropriate. Our reasoning is as follows: An extended version of a low-order graphlet may contain a large number of instances of that low-order graphlet. As an example, a clique of order ten contains (105)=252 instances of *M*_29_. Meanwhile, a clique of order nine contains *only* (95)=126 instances of *M*_29_. Hence, even though only small modifications are made in each step of the generative process, the frequency $C_{M_{29}}$ can, in this case, change by up to a factor of two between two steps. Some other graphlets, most notably *M*_9_, show similar *combinatorial explosions*. We believe a logarithmic contribution is more suitable for capturing this behavior. The base of the logarithm is the factor by which $E_{c}$ must be multiplied to reach $E_{c}\pm 2s_{c}$.

**Algorithm 2. Cost function.**

**Input:** Degree distribution $P_{G}$ and graphlet frequencies $C_{G}$ of graph a *G*, means ${𝐄}_{p}$ and ${𝐄}_{c}$ and standard deviations ${𝐬}_{p}$ and ${𝐬}_{c}$ of degree distribution, respectively graphlet counts, in the target probabilistic network, weight factor w∈[0,1]

**Output:** Cost of graph *G*

1: **Cost**($P_{G}$, $C_{G}$, ${𝐄}_{p}$, ${𝐬}_{p}$, ${𝐄}_{c}$, ${𝐬}_{c}$, *w*)

2: $P_{G}^{c}\leftarrow$ Reversed(CumulativeSum(Reversed($P_{G}$)))

3: ${𝐄}_{p}^{c}\leftarrow$ Reversed(CumulativeSum(Reversed(${𝐄}_{p}$)))

4: $cost_{p}\leftarrow \frac{1}{\left|{𝐄}_{p}\right|}\sum_{i}\left(\frac{\left|P_{G,i}^{c}-E_{p,i}^{c}\right|}{E_{p,i}^{c}}\right)$

5: $cost_{c}\leftarrow 0$

6: **for** $i=1,...,|{𝐄}_{c}|$ **do**

7:   $f\leftarrow \frac{2s_{c,i}}{E_{c,i}}$

8:   **if** $C_{G,i}<E_{c,i}-2s_{c,i}$ **then**

9:    $cost_{c}\leftarrow cost_{c}+\frac{1}{\left|{𝐄}_{c}\right|}{log}_{\left(1-f\right)}\left(\frac{C_{G,i}}{E_{c,i}}\right)$

10:   **else if** $C_{G,i}>E_{c,i}+2s_{c,i}$ **then**

11:    $cost_{c}\leftarrow cost_{c}+\frac{1}{\left|{𝐄}_{c}\right|}{log}_{\left(1+f\right)}\left(\frac{C_{G,i}}{E_{c,i}}\right)$

12:   **end if**

13: **end for**

14: **return** $w\times cost_{p}+(1-w)\times cost_{c}$

Just as in SwapCon, graphlet counts have to be updated in every iteration. However, for GRAIP, this is less of an issue, because changes made to the graph are strictly limited to single nodes or edges. It is now beneficial to use an algorithm that counts graphlets exclusively in the local neighborhood of an added or removed node or edge. ESCAPE cannot easily be adapted to this scenario. Instead, we opt for an enumeration-based approach similar to the IncGraph framework by Cannoodt *et al*. [46], and also adopting some ideas from LINC [33]. We give a brief overview of the approach, but the reader is referred to the above works for more details.

Let’s consider the example of removing an edge $(u,v)\in E_{H}$. The only graphlet instances that can be changed by this operation, are the ones containing (*u*,*v*). Therefore, it is sufficient to enumerate all order $\leq n_{g}$ subgraphs in the depth $\left(n_{g}-2\right)$ neighborhood of (*u*,*v*), i.e. all subgraphs containing *u*, *v* and nodes for which the distance to *u* or *v* is at most $\left(n_{g}-2\right)$. Each thus found subgraph is converted to a bit-string. The nodes of the subgraph are given an arbitrary ordering and a mapping between the node pairs and the positions in the bit-string is constructed as follows: for an order *n*_*S*_ subgraph, a pair $\left(v_{i},v_{j}\right)$, with *i* = 1,..,*n*_*S*_−1, *j* = *i* + 1,...,*n*_*S*_, is mapped to position $\frac{\left(j-1)(j-2\right)}{2}+(i-1)$, where the least significant bit is at position zero [33]. The bit corresponding to pair $\left(v_{i},v_{j}\right)$ is one if $(v_{i},v_{j})\in E_{H}$, and zero otherwise. The list of possible bit-strings per graphlet was precomputed, such that determining the graphlet type of a subgraph only requires looking up the bit-string in a table. The effect of removing an edge is simply a flip of the corresponding bit. The new bit-string might represent a different graphlet, or no graphlet at all, if removing the edge resulted in a disconnected subgraph. This way, it is easy to keep track of the change in graphlet counts. The case of adding an edge is entirely the same. Adding or removing a node is also largely similar. The only differences are that we must enumerate all subgraphs in the depth $\left(n_{g}-1\right)$ neighborhood of the considered node and that graphlet instances can only be created (node added) or destroyed (node removed), but existing ones cannot be changed to another type.

An additional benefit of this approach is that explicitly constructing the temporary graph *T* is actually not necessary. Instead, we simply compute $C_{T}$ and $P_{T}$ based on $C_{H}$ and $P_{H}$ and the modification that would convert *H* to *T*. A modification is only made explicit after it has been accepted. Hence, we prevent copying potentially large graphs.

**Fig 4:**
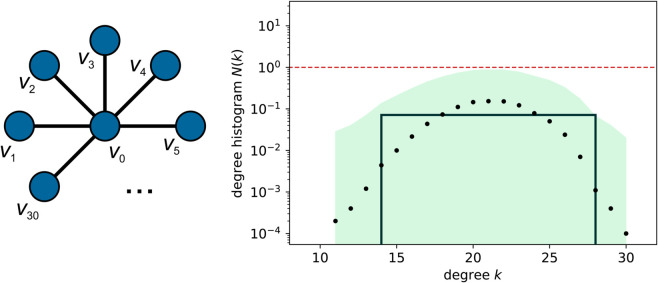
Binning the degree histogram of a 31-node star graph with a uniform edge existence probability of 0.7. The points indicate the mean ${𝐄}_{N}$ and the light green region shows the interval $\left[{𝐄}_{N}\;-\;2{𝐬}_{N},{𝐄}_{N}\;+\;2{𝐬}_{N}\right]$, which lies entirely below the N(k)=1 line. Therefore, the high-degree node would not appear in a generated graph. Applying Algorithm 3 results in a single bin of weight (area) one.

Finally, there is a possible issue with the degree distribution that has so far been ignored. This issue arises if the backbone graph of 𝒢 contains a node of degree *k*, which is substantially different from the degrees of the other nodes. This scenario is not uncommon in biological networks, which sometimes contain one or several nodes of notably higher degree. We illustrate this issue on Fig 4, where we consider the example of a star graph. The broad peak centered at *k* = 21 is actually the result of the single high-degree node of which the degree has been spread out due to the sampling procedure. The degree histogram of a generated graph *H* can never go below the dashed horizontal line, which indicates N(k)=1. Therefore, if $P_{H}$ has to fall within the interval $\left[{𝐄}_{p}-2{𝐬}_{p},{𝐄}_{p}+2{𝐬}_{p}\right]$, or equivalently, $N_{H}$ within $\left[{𝐄}_{N}-2{𝐬}_{N},{𝐄}_{N}+2{𝐬}_{N}\right]$, *H* will never contain the high-degree node found in the real network.

To resolve this issue, degrees are binned in such a way that, for each bin *i* with edges *k*_*i*_ and $k_{i}\;+\;b$, it holds that $\sum_{k\in [k_{i},k_{i}+b)}{𝐄}_{N}[k]\geq 1$. The practical procedure is described in Algorithm 3. Starting from the highest degree found in any of the sample graphs, degrees are taken together in a bin until the weight of the bin is greater than one. The reason for starting at the high-degree side is that isolated peaks are typically found at high degrees, and, since the sum of $E_{N}$-values in a peak must add up to one, we can assure that such peaks are completely included in a single bin. The Boolean variable zero_bin (line 6) keeps track of intervals of *k* that do not occur in any sample and hence should be covered by a bin with weight zero. After defining all bin edges, a final adjustment is made to move long tails of peaks to adjacent bins with weight zero. Otherwise, bins would artificially be widened if the number of samples is increased. We use as a threshold that a certain degree should occur in at least 1% of the samples to be included in a bin with non-zero weight. For bins with width larger than one, the standard deviation on individual points included in the bin is no longer used, as the uncertainty on the number of nodes of degree *k* is already translated to the uncertainty on node degrees through a non-zero bin width. Instead, the $s_{N}$-value is chosen such that the interval $\left[weight\;-\;2s_{N},weight\;+\;2s_{N}\right]$ includes ⌊weight⌋ and ⌈weight⌉, as the weight of a bin is not necessarily an integer, while the number of nodes of *H* that fall in the bin, has to be. For example, if for a certain bin *weight* = 1.6, we set $s_{n}=0.3$.

**Algorithm 3. Bin degree histogram.**

**Input:** Mean degree histogram ${𝐄}_{N}$, number of samples *S*

**Output:** List of bin edges bins, corresponding weights weights

1: **BinDegrees**(${𝐄}_{N}$, *S*)

2: bins← empty list

3: weights← empty list

4: $bin\_edge\leftarrow |{𝐄}_{N}|$

5: Add bin_edge to bins

6: zero_bin← False

7: w←0

8: **while** bin_edge<1 **do**

9:   bin_edge←bin_edge−1

10:    $w\leftarrow w+{𝐄}_{N}[bin\_edge]$

11:    **if** bin_edge=1 **or** w≥1 **or** (zero_bin is True **and** ${𝐄}_{N}[bin\_edge-1]\neq 0$) **then**

12:     Add bin_edge to bins

13:     Add *w* to weights

14:     w←0

15:     $zero\_bin\leftarrow ({𝐄}_{N}[bin\_edge-1]=0)$

16:    **end if**

17: **end while**

18: Adjust bin edges to move tails of peaks where $E_{N}<0.01S$ to neighboring bins with *weight* = 0

19: **return** Reversed(bins), Reversed(weights)

## 4 Numerical experiments

In this section, we evaluate the performance of GRAIP on both synthetic and real network data. In the experiments on real networks, we also make a comparison with SwapCon and two other models from literature: the (dual) BA model [47] and GraphGen [42] (see also Sect 2.2).

*Implementation details.* All our algorithms were implemented in Python using the NetworkX library [48] for graph processing. Our implementations can handle graphlets up to order five and are extendable to larger graphlets. The parameters for the simulated annealing algorithm in SwapCon were taken from [45]: energy threshold of 5%, initial temperature of 0.01 and cooling factor of 0.99. Additionally, the algorithm was stopped if no edge swap had been accepted for $E_{m}$ steps in a row, because the energy threshold of 5% could not always be reached realistically. For the dual BA model, we use the built-in NetworkX function. The dual version was selected for the sole purpose of having more freedom in the number of edges of generated graphs. We pick the number of nodes randomly from a normal distribution with mean $E_{n}$ and variance $s_{n}$, and maintain the same average degree $E_{m}/E_{n}$ as found in the sampled graphs. We use the GraphGen implementation as provided by Goyal *et al*. [42], as well as their proposed hyperparameter settings. Training of GraphGen models was performed on a Linux machine running an AMD EPYC 7413 processor with 12 physical cores at 2.2 GHz and an NVIDIA Ampere A100 GPU with 80 GB GPU memory and 125 GiB RAM. All other experiments were conducted on a single core of an AMD EPYC 7552 processor with 2.6 GiB RAM running at 2.2 GHz.

*Real datasets.* We evaluate our methods on six PPI networks extracted from the IntAct [49], MINT [50] and STRING [51] databases. These sources provide a confidence score on each observed or inferred interaction. More information on these networks is provided in Table 1. For the STRING networks, we explicitly selected only edges with P(e)≥0.7 to obtain examples of high-confidence networks.

**Table 1:** Real PPI networks used in our experiments.

Organism name	Database	Nodes	Edges	$\overset{―}{P(e)}$
*Haloferax volcanii*	IntAct	138	695	0.35
*Escherichia coli*	IntAct	341	682	0.48
*Helicobacter pylori*	MINT	714	1465	0.38
*Saccharomyces cerevisiae*	MINT	4442	18 428	0.46
*Thermococcus nautili*	STRING	158	1275	0.84
*Methanolobus tindarius*	STRING	338	2498	0.79

*Sampling.* In all experiments (synthetic and real data), properties of the target network were derived from 10 000 samples. This is more than enough to ensure convergence. Likewise, GraphGen models were trained on a set of 10 000 graphs sampled from the uncertain network. The running times of the sampling or training algorithms were not included in the computation times reported below, because these only have to be run once if multiple graphs are generated based on the same target network.

### 4.1 Evaluation on synthetic networks

We first evaluate the computation time of GRAIP on synthetic networks of different sizes. The synthetic networks were generated according to the classic ER and BA models, and each edge was assigned a random existence probability, uniformly from (0,1]. For each model and network size, ten uncertain graphs were generated. Then, for each of these, we generated 100 random graphs with GRAIP. The presented running times are hence an average over 1000 runs. At first, we set *n*_*g*_ = 5 and thus consider all 29 graphlets shown on Fig 1. We do not set a maximum number of steps, but keep the algorithm running until the properties of the generated graph lie within the allowed interval.

**Fig 5:**
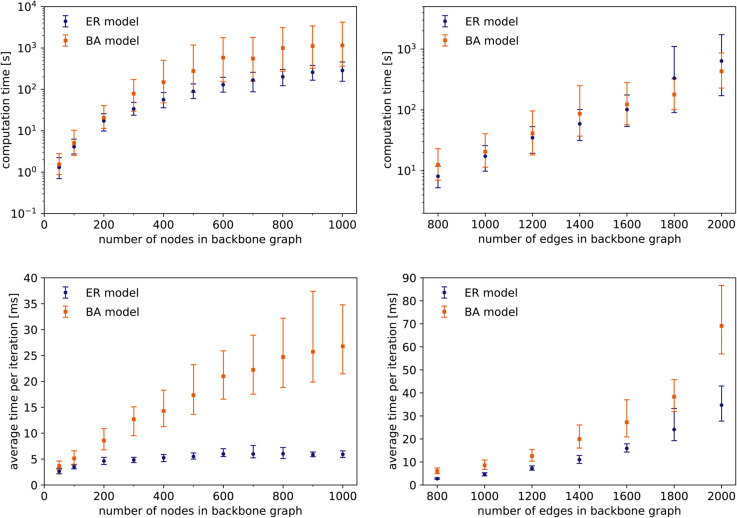
Running time of GRAIP on synthetic probabilistic networks of different order and size. *Left:* Variable order, *m* = 5*n*. *Right:* Variable size, *n* = 200. *Top:* Total time required to generate a graph for which properties lie within the allowed interval. *Bottom:* Average time per iteration (in ms). In all cases, edge probabilities are sampled uniformly from (0,1]. Error bars denote 5th and 95th percentiles.

The influence of the order of the graph is shown on Fig 5, left. The average node degree in the backbone graph is fixed at five. On the top figure, we observe that the computation time increases as the network grows. There is two effects at play here: a larger network requires more iterations of the incremental generation algorithm, and a single iteration requires more computation time. This is why we also show the average time per iteration on Fig 5. Since the computation time of a single iteration is dominated by graphlet counting, the second effect is what we tried to minimize by using an improved counting algorithm. Our method is clearly very effective on ER graphs, as the time per iteration is almost independent of graph order. The effect is less pronounced for BA graphs, although we still observe sublinear scaling with respect to the number of nodes, which is better than the best algorithms that would restart the count from scratch after every adaptation to the graph (see, e.g., [29]). The result is that even graphs with a thousand nodes and several thousand edges can be generated within a reasonable time frame.

Next, we look at the influence of the graph size at fixed order, in this case *n* = 200. The results are shown on the right of Fig 5. At higher graph densities, our counting method is obviously less effective. The “local neighborhood” of a small adaptation to the graph can quickly become the entire network, especially when enumerating five-node graphlets. In such cases, it would be better to use an algorithm not based on enumeration. However, real biological networks, and real-world networks in general, are often rather sparse.

**Fig 6:**
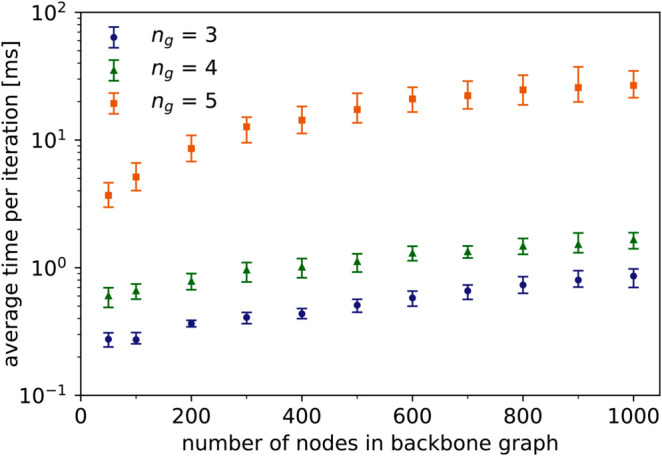
Time per iteration for three values of *n*_*g*_ on synthetic BA networks of different order. Average node degree is fixed at 5 and edge probabilities are chosen uniformly from (0,1]. Error bars denote 5th and 95th percentiles.

Finally, we briefly consider the influence of the *n*_*g*_ parameter, i.e. the maximum order of graphlets taken into account. On Fig 6, we show the time per iteration for different orders of BA graphs and three different values of *n*_*g*_. We do not show the total computation time, because *n*_*g*_ has most effect on the time spent on counting graphlets. The required number of iterations might be reduced significantly as well due to weaker constraints on the generated graphs, but this depends heavily on the topology of the target network. It is clear from Fig 6 that the value of *n*_*g*_ has a big impact on the running time. If only smaller graphlets are considered, the order of graphs that can be generated in reasonable time, can increase by a factor of ten.

### 4.2 Application to real networks

We have demonstrated that GRAIP can effectively generate graphs based on synthetic target networks. However, real networks might have more complicated topological characteristics. We now show the applicability of GRAIP to real PPI networks, and compare its performance to SwapCon and two other models. Before discussing the results, we first explain how we quantified the performance of the different models.

#### 4.2.1 Evaluation metrics.

We want to evaluate the models in terms of both quality and randomness of generated graphs. For the quality, we obviously look at the degree distribution and graphlet frequencies, as these were the characteristics that we wanted to reproduce. An evaluation metric that merely compares average quantities, would not be fitting in the context of uncertain networks. Instead, we use the metric proposed in [41], which includes information on the distribution of quantities. We give a brief overview below.

The metric is based on the Maximum Mean Discrepancy (MMD), computed using a certain graph statistic. The MMD requires a choice of kernel function. A common choice is the Gaussian kernel:

$$k(x,y)=\exp\left(-\frac{\|x-y{\|}^{2}}{2\sigma^{2}}\right).$$ (8)

Here, x and y are vectors of sampled statistics and *σ* is the bandwidth parameter. Based on this kernel, the squared MMD is computed as

$${MMD}^{2}=\;\frac{1}{\left|x|(|x|-1\right)}\sum_{i\neq j}k(x_{i},x_{j})\;+\frac{1}{\left|y|(|y|-1\right)}\sum_{i\neq j}k(y_{i},y_{j})-\frac{2}{\left|x||y\right|}\sum_{i,j}k(x_{i},y_{j}).$$ (9)

The (squared) MMD is minimal if the distributions of x and y are identical. In our case, the graph statistic is either the degree distribution or the graphlet counts. The vector x contains, e.g., graphlet counts obtained from a set of sample graphs, while y contains counts obtained from a set of newly generated graphs.

Our objective was to generate graphs with degree distributions and graphlet frequencies similar to an uncertain network, but that are random in other aspects. Quantifying randomness in a collection of graphs is not an easy task. In this work, we restrict ourselves to determining the spread on two graph properties that are not directly controlled by any of the considered models: the diameter and the average local clustering coefficient. The diameter of a graph is defined as the longest of the shortest paths between all pairs of nodes. The local clustering coefficient $CC_{l,v}$ was defined in Eq 2. This quantity is averaged over all nodes to obtain $\overset{―}{CC_{l,v}}=\frac{1}{n}\sum_{v\in V}CC_{l,v}$. We compute both properties in all generated graphs and define the spread as the difference between the 5th percentile and the 95th percentile, to exclude rare outliers. This is compared to the spread found in a set of sample graphs and we report the ratio between the two as a measure for randomness. A ratio above or below one means there is more, respectively less, variety in the generated graphs than in the sample graphs, at least with respect to the considered property.

#### 4.2.2 Results and discussion.

For each dataset, we generated 1000 graphs with every model. GraphGen model training on the *S. cerevisiae* and *M. tindarius* networks could not be completed in a reasonable time. After three days of training, we still had not completed 1000 epochs, and the other networks required well over 10 000 epochs to minimize the loss. This illustrates the poor scaling of machine learning models mentioned previously. Unlike in the experiments on synthetic networks, we now give a maximum number of steps to GRAIP, equal to 100 times the expected number of edges $E_{e}$. Additionally, all models were halted if graph generation took longer than one hour, but this only happened for SwapCon on two of the datasets. In this case, we saved the best graph constructed so far. We now only consider graphlets up to order four. We could easily go up to order five on the smaller networks, at least with GRAIP, but we restrict this evaluation to order four on all networks for consistency.

**Fig 7:**
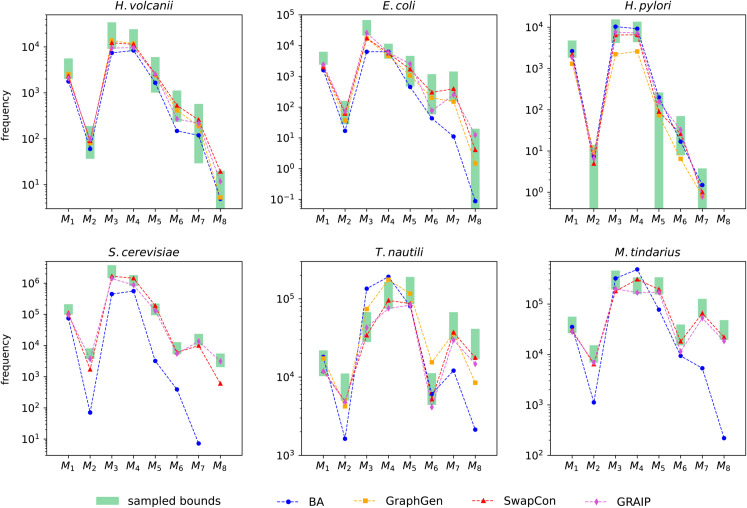
Average frequencies of 3- and 4-node graphlets in generated graphs. The green bands indicate the uncertainty intervals derived from sampling the real network (mean plus/minus two standard deviations).

First, we provide a qualitative evaluation of the generated graphs. Fig 7 shows the average graphlet frequencies in generated graphs and the bounds derived from the target network. The graphlet frequencies of SwapCon and GRAIP graphs consistently lie within, or very close to, the bounds. Significant deviations occur in GraphGen and, in particular, BA graphs, especially on the larger and denser networks (bottom row). It should be noted that on the *S. cerevisiae*, *T. nautili* and *M. tindarius* networks, GRAIP ran into the step limit for over 95% of the generated graphs. Still, only a small deterioration in performance is noticeable. This indicates that GRAIP can still produce a decent graph if the algorithm is stopped early, thanks to the incremental generation approach.

Numerical results are presented in Table 2. To compute the ${MMD}^{2}$ scores, generated graphs were split in ten batches of 100 graphs each, and the reported score is the average over the ten batches. In terms of quality metrics, SwapCon excels at reproducing the statistics of the degree distribution. This was to be expected, as the degree sequence is extracted directly from the sampled degree distribution and node degrees cannot change by performing edge swaps. GRAIP still shows good performance, comparable to GraphGen. The BA model notably performs far worse, even though the degree distributions of most considered networks (less so for the two STRING networks) closely follow power laws. Furthermore, GRAIP scores best on graphlet frequencies on four of the six networks. We see again that GRAIP produced graphs in excellent agreement with the *S. cerevisiae*, *T. nautili* and *M. tindarius* networks, despite the algorithm being halted early by the step limit. The fact that GraphGen shows by far the best performance on the *H. volcanii* network is another indication that machine learning models are currently mostly suited for relatively small networks. Moreover, note that our MMD metric actually favors GraphGen, because neither SwapCon, nor GRAIP, were designed to reproduce the statistical distribution of, e.g., graphlet counts across the different samples. SwapCon targets the mean and GRAIP only ensures that counts lie within certain margins. GraphGen models obtain all information about the distribution during training, yet we still observe that our models perform at least as well in most cases.

**Table 2:** Performance of the (dual) BA model, GraphGen, SwapCon and GRAIP on six PPI networks.

Dataset	Model	Quality		Randomness		Running
		Degree ($\times 10^{-4}$)	Graphlets	Diameter	$\overset{―}{CC_{l,v}}$	Time [s]
*H. volcanii*	BA	11.9	0.97	0.00	1.20	≪1
	GraphGen	0.10	0.07	1.00	0.97	25.4
	SwapCon	0.01	0.28	4.00	1.65	78.9
	GRAIP	0.45	0.90	1.00	0.80	7.3
*E. coli*	BA	1.66	1.27	0.33	0.47	≪1
	GraphGen	0.17	0.55	2.00	1.43	26.9
	SwapCon	≈0	0.49	2.83	1.42	27.1
	GRAIP	0.16	0.35	0.50	0.85	20.5
*H. pylori*	BA	0.04	0.65	0.40	1.66	≪1
	GraphGen	0.36	1.06	1.20	3.45	27.5
	SwapCon	≈0	0.22	1.20	0.88	17.1
	GRAIP	0.05	0.54	0.60	2.44	4.1
*S. cerevisiae*	BA	0.27	1.55	0.00	0.24	≪1
	SwapCon	≈0	0.72	1.00	0.69	>1h
	GRAIP	0.01	0.47	0.67	6.58	3491
*T. nautili*	BA	6.63	0.743	0.33	0.78	≪1
	GraphGen	0.42	0.61	1.67	1.91	28.1
	SwapCon	0.01	0.63	1.00	2.07	1243
	GRAIP	1.51	0.61	1.33	2.64	541
*M. tindarius*	BA	2.57	0.83	0.00	0.49	≪1
	SwapCon	≈0	0.61	0.67	2.01	>1h
	GRAIP	0.17	0.48	1.33	4.23	1145

Quality metrics are the ${MMD}^{2}$ scores computed as an average of ten comparisons between 100 generated graphs and 100 sampled graphs. The degree score was multiplied by 10^4^. Randomness is quantified by the relative spread. For more information on these metrics, see the main text. Running time of BA graph generation is in the order of milliseconds or less, and is therefore reported as “≪1". If graph generation ran into the one hour time limit, we report “>1h”.

The BA model is generally worst in terms of randomness, but there is no model that is clearly better than all others. Even graphs produced by GraphGen models are often more diverse than the training set. We observe that GRAIP shows less diversity on the smaller, sparse networks. These networks are unlikely to contain complicated topological characteristics and, therefore, relatively few iterations are required to obtain a graph with suitable properties. It might be better in these cases not to stop the algorithm immediately when an acceptable graph is obtained, but to allow more modifications for further randomization.

The benefit of the simplicity of classical models like the BA model, is that generation of graphs with thousands of nodes and edges is almost instant. Graph generation with GraphGen is fast as well and, unlike model training, does not seem to depend much on the size of the graph. However, it is worth noting that training already took several days on the smallest networks considered here. Even without taking training time into account, GRAIP beats GraphGen in terms of computation speed, except on the high-confidence *T. nautili* network, and is up to ten times faster than SwapCon.

## 5 Conclusion

In this paper, we have examined the generation of random graphs with graphlet frequencies and degree distribution prescribed by a probabilistic target network. Probabilistic networks are ubiquitous in the real world, especially in biology, but the uncertainty is often overlooked. We have shown that the properties of possible graphs sampled from a probabilistic network can differ greatly from those of the backbone graph, obtained by omitting all information about uncertainty. We have extended the swapping model for deterministic graphs to SwapCon, a model applicable to uncertain graphs, and introduced a novel algorithm, GRAIP, to generate graphs incrementally. GRAIP improves upon SwapCon by making better use of the statistical information on graph properties. On top of that, the incremental algorithm allows for a more efficient method of counting graphlets and considerably increased computation speed. Our algorithm scales well to larger networks, as long as the networks are sparse, which is usually the case in biology. Moreover, on large, dense networks, the graphlet counting problem itself is computationally intractable, at least with the currently available methods.

An important restriction of this work is that we have only considered simple, undirected graphs. In future work, we may extend our model to more diverse networks, with different types of nodes and edges. The key challenge here is that this will drastically increase the number of graphlet types. For example, just adding a direction to edges already increases the number of unique three-node graphlets to 13, and the number of unique four-node graphlets to 218. This not only affects the complexity of counting all small patterns, but also puts significantly more constraints on the graphs to be generated. Therefore, the problem only seems tractable if the graphlet frequencies of a small subset of graphlets is monitored. In this extended model, a practitioner would be able to select some graphlet topologies which they deem most interesting and of which the frequencies would be reproduced in the generated graphs, while frequencies of other graphlets are allowed to fluctuate freely.
